# Correspondence

**Published:** 1875-05

**Authors:** C. W. Spalding


### Correspondence,

EDITOR DENTAL REGISTER.

I am glad to see that the question of the relation which dentistry holds to medicine is being discussed in your columns. I am decidedly of the opinion that the question is one of too much importance to longer remain undetermined. As an intelligent body, we have occupied an equivocal position quite long enough. Let us then give the matter that deliberate consideration which its great importance demands; and having done this in a thorough, careful aud impartial manner, let us so fully, and so clearly define our position, that there shall hereafter be no uncertainty about our professional status.

Dr. Allport's proposition to separate the departments of dentistry or at least to drop one branch that is now recogized as legitimate and place it in a separate and independent position has had the effect of opening discussion on the main question at issue, viz: "What relation does dentistry hold to medicine." For that service the Dr. deserves the thanks of the profession.

I have long regarded this question as a pending one, and the current of events has not encouraged me to hope that it was one that would peaceably adjust itself. It so nearly concerns our future, that I think every one will acknowledge the expediency, if not the necessity of dealing with it at an early day.

Our educational system, though somewhat diversified at present, is fast assuming more definite proportions and will at no distant day crystallize into some permanent form, possessing that coherence and uniformity which should always characterize a specific educational system. What the character of that form shall be it is not our province to decide. It is evident that dentistry must either constitute an independent profession, or become a specialty of medicine.

The former implies independent schools, requiring text books, adapted to the system of education which such schools would develop, and which with a few commendable exceptions are as yet unwritten. The latter calls for a medical education in conformity with a system already established by the medical profession, to which must be added a special education in dentistry by means of schools organized for that specific purpose.

In all the specialties of medicine a thorough medical education is the first requisite. The candidate becomes first a physician, and afterwards a specialist. The rule would continue to be imperative, and would apply to dentistry equally with other specialties. So comprehensive a course of study would necessarily imply a longer pupilage than has heretofore been customary with dental students and would tend to diminish the number of candidates.

An improved dental education is a confessed want, and I consider that as a first condition of its attainment, the status of dentistry as a profession, shall we accurately determined. This point once settled our course would be plain and we could all work together for the accomplishment of a common object. Now one part is working for independent colleges, while another favors combined schools, or rather special schools attached to medical colleges.

The general principles of medicine ( I use the term in its broadest sense) would be acquired in a medical school and would serve as an excellent basis for a subsequent special course in Dentistry. This I think no one will dispute. But the special course will be absolutely necessary to the proper qualification of the dental practitioner. I do not think this second postulate will be any more questioned than the former. Two collegiate courses will therefore be required.

The chief consideration which at this point forces itself upon our attention is this. Is it practicable, to effect so important a change in our prevalent educational system? The excellence of the results which would follow the enforcement of such a rule, can not be questioned. By its operation dentistry would, so far as education is concerned, be elevated to a much higher position, than it has hitherto attained.

I am decidedly favorable to an advanced system of education and shall not be critical of the mode provided it is acceptable to the great body of the profession. Any scheme therefore that can be made effective for the improvement of the general qualifications of the dentist will receive my most cordial support. I here distinguish between general and special qualifications. The facilities for obtaining the latter are already sufficient for our present wants and it is to the former that our attention should now be directed.

It would be manifestly unwise to introduce any custom or rule that did not promise to become universal; and, hence, in my estimation, the whole matter turns upon the question of the relative practicability of the methods proposed.

The main object of this communication is to elicit the views of others upon the question under discussion, and I have therefore in this article endeavored to state the question fairly, reserving its consideration more in detail until it shall appear that the profession are ready for "a new departure."

C. W. Spalding.

